# Comparative Performance of Ground vs. Aerially Assessed RGB and Multispectral Indices for Early-Growth Evaluation of Maize Performance under Phosphorus Fertilization

**DOI:** 10.3389/fpls.2017.02004

**Published:** 2017-11-27

**Authors:** Adrian Gracia-Romero, Shawn C. Kefauver, Omar Vergara-Díaz, Mainassara A. Zaman-Allah, Boddupalli M. Prasanna, Jill E. Cairns, José L. Araus

**Affiliations:** ^1^Integrative Crop Ecophysiology Group, Plant Physiology Section, Faculty of Biology, University of Barcelona, Barcelona, Spain; ^2^International Maize and Wheat Improvement Center, CIMMYT Southern Africa Regional Office, Harare, Zimbabwe

**Keywords:** maize, remote sensing, UAV, RGB Vis, multispectral Vis, phosphorous fertilization

## Abstract

Low soil fertility is one of the factors most limiting agricultural production, with phosphorus deficiency being among the main factors, particularly in developing countries. To deal with such environmental constraints, remote sensing measurements can be used to rapidly assess crop performance and to phenotype a large number of plots in a rapid and cost-effective way. We evaluated the performance of a set of remote sensing indices derived from Red-Green-Blue (RGB) images and multispectral (visible and infrared) data as phenotypic traits and crop monitoring tools for early assessment of maize performance under phosphorus fertilization. Thus, a set of 26 maize hybrids grown under field conditions in Zimbabwe was assayed under contrasting phosphorus fertilization conditions. Remote sensing measurements were conducted in seedlings at two different levels: at the ground and from an aerial platform. Within a particular phosphorus level, some of the RGB indices strongly correlated with grain yield. In general, RGB indices assessed at both ground and aerial levels correlated in a comparable way with grain yield except for indices a^*^ and u^*^, which correlated better when assessed at the aerial level than at ground level and Greener Area (GGA) which had the opposite correlation. The Normalized Difference Vegetation Index (NDVI) evaluated at ground level with an active sensor also correlated better with grain yield than the NDVI derived from the multispectral camera mounted in the aerial platform. Other multispectral indices like the Soil Adjusted Vegetation Index (SAVI) performed very similarly to NDVI assessed at the aerial level but overall, they correlated in a weaker manner with grain yield than the best RGB indices. This study clearly illustrates the advantage of RGB-derived indices over the more costly and time-consuming multispectral indices. Moreover, the indices best correlated with GY were in general those best correlated with leaf phosphorous content. However, these correlations were clearly weaker than against grain yield and only under low phosphorous conditions. This work reinforces the effectiveness of canopy remote sensing for plant phenotyping and crop management of maize under different phosphorus nutrient conditions and suggests that the RGB indices are the best option.

## Introduction

Sub-Saharan Africa (SSA) has one of the world's fastest growing populations but the growth rate of food production has not kept pace with this, leading to a food deficit (Mclntyre et al., 2009). Low levels of soil phosphorous (P) and nitrogen (N), are the main constraints to crop growth in these areas (Buerkert et al., 2001). Phosphorous fertilizers are relatively costly in SSA and are scarce in some countries, partly due to poorly developed markets, and so phosphorous application is low (1 kg ha^−1^ compared with 14.3 kg ha^−1^ in Asia) (Smalberger et al., 2006). Plant scientists face the challenge of solving these limitations while taking into account the additional implications of climate change on food security (Cairns et al., 2012, 2013a). In that sense, affordable technologies capable of monitoring crop performance for agronomical purposes, yield prediction or to assess phenotypic variability for breeding are bottlenecks in the pathway to full exploitation of this technology (Reynolds et al., 2012; Araus and Cairns, 2014).

Remote sensing has become an important methodology for the application of agricultural monitoring and to improve precision and throughput in phenotyping. There is a growing body of literature demonstrating the usefulness of remote sensing for a wide range of applications in agriculture: growth monitoring, yield prediction, stress detection, nutrient deficiency diagnosis, and control of plant diseases (Fiorani and Schurr, 2013). In the case of phenotyping, these methodologies offer the opportunity to screen large numbers of genotypes at a lower cost and faster than conventional phenotyping and provide to breeding programs the opportunity to assess genetic diversity under field conditions. Remote sensing methods enable detailed non-invasive information to be captured throughout the plant life cycle. Among the different remote sensing techniques, the most usual indices used are derived from Red-Blue-Green (RGB) images (Casadesús et al., 2007) and multispectral (Thenkabail et al., 2002), hyperspectral (Blackburn, 2007) and thermal sensors and images (Araus and Cairns, 2014; Deery et al., 2016). However, large differences exist in the price of the different equipment deployed (e.g., spectrometers vs. conventional red/green/blue cameras).

The traditional procedure has involved the use of multispectral sensors and the development of numerous vegetation indices associated with vegetation parameters such as above-ground biomass, water and nutrient deficiency, and crop yield (Petropoulos and Kalaitzidi, 2012). The Normalized Difference Vegetation Index (NDVI) is one of the most well-known vegetation indices derived from multispectral remote sensing, as it includes visible and near infrared radiation. Although, it was originally developed for satellite remote vegetation sensing, it has also been found useful in ground-based and aerial applications. In fact, several groups of spectral variables have been identified as being of value in characterizing plant performance and empirical indices have been defined. Among these, some are modifications of the NDVI that takes atmospheric effects and/or soil influences into account in order to increase their sensitivity, like the Soil-adjusted Vegetation Index (SAVI) or the Renormalized Vegetation Index (RDVI) (Wu, 2014). Others, like the Photochemical Reflectance Index (PRI), aim to assess how efficiently the radiation is used by plants during photosynthesis, while the Modified Chlorophyll Absorption in Reflectance Index (MCARI) or the Transformed Chlorophyll Absorption in Reflectance Index (TCARI) (Haboudane et al., 2002), are focused on quantifying photosynthetic pigments. Further, other indices also have been used to determine the water status of plants, like the Water Index (WI) (Peñuelas et al., 1993; Babar et al., 2006).

The use of information derived from conventional digital RGB (of red, green, blue) images may represent a low-cost alternative to the use of multispectral or hyperspectral information for formulating vegetation indices. The images can be processed to convert RGB values into indices based on the models of Hue-Intensity-Saturation (HIS), CIELab, and CIELuv cylindrical-coordinate representations of colors. The RGB indices implementation has been extensive and successful for providing a wide-range of phenomic data about genotypic performance under different growing conditions (Casadesús et al., 2007; Casadesús and Villegas, 2014; Vergara-Díaz et al., 2015, 2016; Zaman-Allah et al., 2015; Zhou et al., 2015; Yousfi et al., 2016).

The environmental variability throughout the day, like changes in radiation, temperature or the occurrence of clouds, affects the phenotypic observations inconsistently and may limit the accuracy of the time-consuming proximal measurements at ground level (e.g., the relative chlorophyll content). The incorporation of these methodologies into aerial based platforms enables the simultaneous characterization of a larger number of plots (i.e., spectral reflectance at solar noon), which may help to minimize the effect of changing environmental conditions (Araus and Cairns, 2014). This becomes extremely important with regards to the increasing demand to support and accelerate progress in breeding for novel traits, which at the same time requires accurate high throughput phenotyping of a large numbers of plants. Furthermore, the added cost of the aerial platforms may be offset by time savings by reducing manual field labor.

The vegetation indices, formulated from the visible and infrared spectrum of the light reflected by plants or derived from RGB conventional digital images are the most usual remote sensing method to assess plant nutrient status (Vergara-Díaz et al., 2016). However, while most studies that have focused on the spectral evaluation of nutrient deficiencies of crops have concerned analysis of nitrogen content, such evaluations are far less common with other nutrients, including phosphorous (Osborne et al., 2002; Mahajan et al., 2014). In addition to the reduction in the total biomass, the lack of other mineral nutrients can also influence the color of leaves. In the case of phosphorus, it is well-known that leaf darkening is caused by a phosphorous deficiency, but the relationship between symptoms and leaf color is less evident than for nitrogen deficiency.

Because maize is among the major crops globally, and the main staple for direct human consumption in SSA (Cairns et al., 2013b), the aim of this study was to test the efficiency of different remote sensing methods and tools in assessing the yield performance and the phosphorus status of a total of 26 maize hybrids under optimum and no phosphorus fertilization. The performance of remote sensing assessment from an unmanned aerial platform and from the ground was compared. Different categories of sensors were tested, including RGB cameras (placed on an aerial platform as well as at ground level), alongside a multispectral camera (on the aerial platform) and a spectrometer with an active sensor designed to measure the NDVI at ground level. Measurements were performed at the seedling stage in order to assess early predictions of plant performance and yield. Phosphorus fertilization affects plant growth which subsequently may alter water status (e.g., through differences in the amount transitive area or in root development) and nitrogen uptake and assimilation. In that sense, the stable isotope compositions of C and N (δ^13^C and δ^15^N) were measured in leaf samples as a complementary selection traits, aiming to assess any effect of phosphorous assimilation on the water status and nitrogen metabolism of the plant. Thus, for a C4 species such as maize in spite δ^13^C composition while barely reflects genotypic variability in water performance, it may still catch differences between treatments in the plant water status (Cabrera-Bosquet et al., 2009); while δ^15^N may reflect the effect of the treatment on the uptake and further assimilation of N (Evans, 2001).

## Materials and methods

### Plant material and growing conditions

Field trials were carried out at the Southern Africa regional station of CIMMYT (International Maize and Wheat Improvement Center) located in Harare (−17.800, 31.050, 1498 masl), Zimbabwe. The soil in the station is characterized by a pH slightly lower than 6, nitrogen as nitrate (${\text{NO}}_{3}^{-}$) of around 4 μg g^−1^ and phosphorous contents of nearly 20 μg g^−1^ (Vergara-Díaz et al., 2016).

A set of 25 maize hybrids developed at CIMMYT plus a local check (CZH131001, CZH0524, CZH141042, CZH0631, CZH131002, CZH0513, CZH131007, CZH03042, CH12716, CZH03004, CZH15020, SC513, CZH132210, CZH142125, CZH132218, CZH142153, CZH142159, SC719, CZH142186, CZH142212, CZH142074, CZH142003, CZH142206, CZH142195, and CZH142210) were sown during the wet season on December 2015. These maize hybrids reflect a large variability in plant performance to different phosphorous conditions. The experimental design consisted of two separated phosphorous treatments with 26 plots each corresponding to each maize genotype studied (52 plots in total).

Seeds were planted on December 21^st^ 2015, in three rows per plot; rows were 4 m long and 75 cm apart (9 m^2^/plot), with 17 plants per row and 25 cm between plants in each a row. A split-plot in a randomized complete block design without replicates was used. The field was fertilized with 200 kg·ha^−1^ of ammonium nitrate (AN) and 250 kg·ha^−1^ of muriate of potash before sowing (basal fertilizer), followed with 250 kg·ha^−1^ AN for top dressing. In order to generate differential phosphorus conditions, 400 kg/ha of superphosphate fertilizer were added at pre-sowing to one half of the trial, corresponding to the optimum phosphorous fertilized conditions (OP). The other part of the trial corresponded to the non-phosphorus fertilized conditions (NPF). The trial was depleted of phosphorus for 1 year. A two-row border of a commercial maize variety was sown on the edges of the trial to prevent border effects. Trials were gathered following the standard procedures of CIMMYT. The central 3.5 m of each row was harvested discarding 2 plants at each end, thus the collected grain yield (t·ha^−1^) corresponded to the weight of 7.87 m^2^.

In addition, these hybrids were also tested in other trials in Zimbabwe under optimal fertilization conditions comparable to those of the OP trial of the experimental station. Evaluations were performed at the Agricultural Research Trust site in Harare (−17.716, 31.716, 1,516 masl). For these trials, the fertilization conditions were basically the same than at the OP conditions of the main study (CIMMYT Station).

### Proximal and aerial data collection

Remote sensing evaluations were performed on seedlings (<5 leaves) during the last week of January. Vegetation indices derived from RGB images were evaluated for each plot at ground and aerial levels. At ground level one conventional digital picture was taken per plot, holding the camera about 80 cm above the plant canopy in a zenithal plane and focusing near the center of each plot. The digital camera used was an Olympus OM-D (Olympus, Tokyo, Japan). Pictures were acquired at a 16-megapixel resolution with a sensor using a 14-mm focal length, triggered at a speed of 1/125 s with the aperture programmed in automatic mode. NDVI was also determined on individual plots at ground level using a portable spectrometer (GreenSeeker handheld crop sensor, Trimble, USA). Additionally, the leaf chlorophyll content (LCC) of the last developed leaf was measured using a Minolta SPAD-502 portable chlorophyll meter (Spectrum Technologies Inc., Plainfield, IL, USA). Eight leaves were measured for each plot (four per row), each leaf being the last fully expanded within a plant. For each leaf four measurements were taken from the middle portion of the lamina.

**Figure 1:**
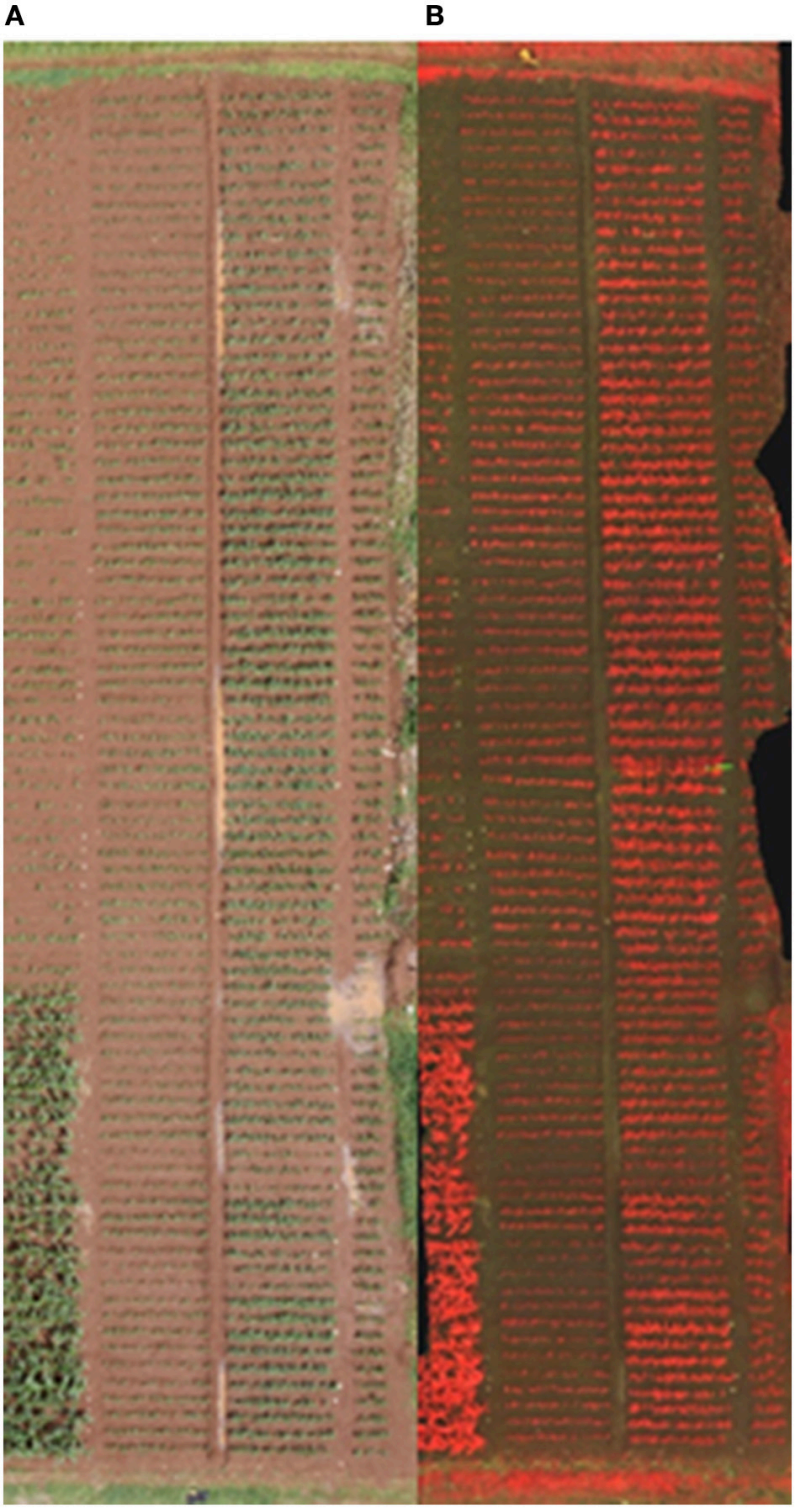
RGB **(A)** and false-color infrared **(B)** ortho-mosaics of the plot images under P fertilization (right plots) and no fertilization (left plots).

Further, RGB and multispectral aerial images were acquired using an unmanned aerial vehicle (UAV) (Mikrokopters OktoXL, Moormerland, Germany) flying under remote control at around 50 m (Figure 1). The camera used for the aerial images was a Lumix GX7 (Panasonic, Osaka, Japan), a digital single lens mirrorless camera with an image sensor size of 17.3 × 13.0 mm. Images were taken at 16-megapixel resolution using a 20-mm focal length. In addition, a multispectral camera covering wavelengths in the visible and near infrared regions of the spectrum (MCA12, Tetracam Inc., Chatsworth, CA, US) was also mounted in the drone. The camera consisted of 12 independent image sensors, and optics with user configurable filters. It captured 15.6-megapixels of image data and transferred this to 12 separate flash memory cards. Both RGB and multispectral images were taken at the rate of one every 5 s.

### Image processing

To obtain correct image mosaics from the multispectral images a 3D reconstruction approach was needed to produce an accurate ortho-mosaic and remove the effects of the UAV flight. Agisoft PhotoScan Professional (Agi- soft LLC, St. Petersburg, Russia) was employed for this task using 20–30 overlapping images for both mosaics (RGB and multispectral) with at least 80% overlap. Through the open source image analysis platform FIJI (Fiji is Just ImageJ; http://fiji.sc/Fiji), regions of interest were established at each row for the plots to be cropped.

RGB pictures were subsequently analyzed using a version of the Breedpix 0.2 software adapted to JAVA8 and integrated as a plugin within FIJI; https://github.com/George-haddad/CIMMYT). This software enables the extraction of RGB vegetation indices (VIs) in relation to different properties of color (Casadesús et al., 2007). Essentially, the indices are based on either the average color of the entire image, in diverse units related to its “greenness,” or on the fraction of pixels classified as green canopy relative to the total number of pixels in the image. In HSI color space, the Hue (H) component describes the color itself traversing the visible spectrum in the form of an angle between 0° and 360°, where 0° means red, 60° means yellow, 120° means green and 180° means cyan. Derived from the Hue, Green Area (GA), and Greener Area (GGA) analyze the proportion of green pixels in the image. GA is the percentage of pixels in the image in the hue range from 60 to 180°, that is, from yellow to bluish green. Meanwhile, GGA is somewhat more restrictive because the range of hue considered by the index is from 80 to 180°, excluding yellowish-green tones. In the CIELab color space model, dimension L^*^ represents lightness, and the green to red range is expressed by the a^*^ component, with a more positive value representing a purer red, and conversely a more negative value indicating a greener color. Meanwhile, blue to yellow is expressed by the b^*^ component, where the more positive the value the closer it is to a pure yellow, whereas the more negative the value the closer it is to blue. Similarly, in the CIELuv color space model, dimensions u^*^ and v^*^ are perceptually uniform coordinates, where the visible spectrum starts with blue at the bottom of the space, moving through green in the upper left (mostly scaled by v^*^) and out to red in the upper right (mostly scaled by u^*^). The multispectral indices, formulated with the Tetracam camera and detailed in Table 1, were calculated from the multispectral images using a custom FIJI macro code.

**Table 1:** Indices derived from the multispectral visible and near infrared bands.

**Target group**	**Index**	**Equation**	**Wavelengths**	**References**
Broadband greenness	Normalized difference vegetation index (NDVI)	(B840 – B670)/(B840 + B670)	Red, NIR	Rouse et al., 1973
	Soil adjusted vegetation index (SAVI)	(B840 – B670)/(B840 + B670 + L)^*^(1 + L)	Red, NIR	Huete, 1988
		Low vegetation, L = 1, intermediate, 0.5, and high 0.25		
	Optimized soil-adjusted vegetation index (OSAVI)	((1 + 0.16)^*^(B780 – B670))/((B780 + B670 + 0.16))	Red, NIR	Rondeaux et al., 1996
	Renormalized difference vegetation index (RDVI)	(B840 – B670)/((B840 + B670)∧1/2)	Red, NIR	Roujean and Breon, 1995
	Enhanced vegetation index (EVI)	2.5^*^(B840 – B670)/(B840 + (6^*^B670) − (7.5^*^B450) + 1)	Blue, Red, NIR	Huete et al., 2002
Light Use efficiency	Photochemical reflectance index (PRI)	(B550 – B570)/(B550 + B570)	Green	Gamon et al., 1997
Leaf pigments	Modified chlorophyll absorption ratio index (MCARI)	[(B700 – B670) – 0.2^*^(B700 – B550)]^*^B700/B670	Green, Red	Daughtry, 2000
	Transformed chlorophyll absorption in reflectance index (TCARI)	3^*^(B700 – B670)-0.2^*^(B700 – B550)^*^(B700/B670)	Green, Red, NIR	Haboudane et al., 2002
	Anthocyanin reflectance index 2 (ARI2)	B840^*^(1/B550 – 1/B700)	Blue, Red, NIR	Gitelson et al., 2001
	Carotenoid reflectance index 2 (CRI2)	1/B550 – 1/B700	Blue, Red	Gitelson et al., 2002
Canopy water content	Water band index (WBI)	(R840 – B670)/(B840 + B670)∧(1/2)	Red, NIR	Peñuelas et al., 1993

### Leaf phosphorous content

Similar leaves to those used for leaf chlorophyll measurements were sampled and subsequently oven dried at 70°C for 24 h and ground to a fine powder. For the analysis of P content, a total of 100 mg of sample were digested in acid for 24 h at 90°C within Teflon vessels, using 2 ml of NHO_3_ and 0.5 ml of hydrogen peroxide, with samples subsequently re-suspended in 30 ml of deionized water. Analyses were performed by Inductively Coupled Plasma Optical Emission Spectroscopy (ICP-OES) using a Perkin-Elmer Optima 3200RL Spectrometer (Perkin-Elmer, Massachusetts, EEUU) at the Scientific Facilities of the University of Barcelona. Leaf phosphorous content was expressed in mg of P per g of dry mass.

### Total nitrogen content and carbon and nitrogen stable isotope compositions

The same ground material was also used to analyze the total nitrogen content together with the stable isotopic abundances of carbon and nitrogen in the leaves. Samples of about 0.7 mg of dry matter and reference materials were weighed into tin capsules, sealed, and then loaded into an elemental analyzer (Flash 1112 EA; ThermoFinnigan, Schwerte, Germany) coupled with an isotope ratio mass spectrometer (Delta C IRMS, ThermoFinnigan), operating in continuous flow mode. Measurements were carried out at the Scientific Facilities of the University of Barcelona. The ^13^C/^12^C ratios (R) of plant material were expressed in composition (δ^13^C) notation (Coplen, 2008) as follows:

(1) $$\delta^{13}\text{C}\;(‰)=[(\text{R sample}/\text{Rstandard})\;-1]\;x1000$$

Where: sample refers to plant material and standard to Pee Dee Belemmite (PDB) calcium carbonate. International isotope secondary standards of a known ^13^C/^12^C ratio (IAEA CH7, polyethylene foil, IAEA CH6 sucrose and USGS 40 l-glutamic acid) were calibrated against Vienna Pee Dee Belemnite calcium carbonate (VPDB) with an analytical precision of 0.1%0. The ^15^N/^14^N ratios of plant material were also expressed in δ notation (δ^15^N) using international secondary standards of known ^15^N/^14^N ratios (IAEA N1 and IAEA N_2_ ammonium sulfate and IAEA NO_3_ potassium nitrate), with analytical precision of about 0.2%0. Further, the C/N ratio was obtained from these analyses.

### Statistical analysis

Statistical analyses were conducted using the open source software, RStudio 1.0.44 (R Foundation for Statistical Computing, Vienna, Austria). Data for the set of physiological traits were subjected to factorial analyses of variance (ANOVAs) to test the effects of growing conditions on the different traits studied. A bivariate correlation procedure was used to calculate the Pearson correlation coefficients of the different remote sensing indices against the grain yield and the leaf phosphorus content. Multiple regressions were calculated via a forward stepwise method with GY and P content as dependent variables and the different indices as independent parameters. The figures were also drawn using the Rstudio software.

## Results

### The effect of phosphorous availability on grain yield and leaf parameters

Omission of phosphorous fertilizer significantly decreased yield from a mean value (across genotypes) of 7.50 to 5.64 t ha^−1^ under optimum and no-phosphorous fertilizer conditions, respectively (Table 2). Moreover, the varieties presented a wide range of yield and leaf phosphorus content within the fertilization conditions. Despite this, the phosphorus content of the leaves only correlated significantly against grain yield under non-phosphorus-fertilized conditions (Supplementary Figure 1).

**Table 2:** Effect of supplemental phosphorus fertilization on the grain yield (GY), leaf chlorophyll content (LCC), phosphorous content (P), leaf carbon and nitrogen concentration (C and N), leaf C/N ratio, and the stable carbon (δ^13^C) and nitrogen (δ^15^N) composition within the non-phosphorous fertilized (NPF) and the optimal phosphorous (OP) conditions.

	**NPF**	**OP**	***p*-value**
GY (t ha^−1^)	5.64 ± 0.20	7.5 ± 0.20	0.000^***^
LCC	32.01 ± 0.99	46.19 ± 0.78	0.000^***^
P (mg/g DW)	2.06 ± 0.08	4.81 ± 0.11	0.000^***^
C (%)	43.62 ± 0.10	43.03 ± 0.23	0.021^*^
N (%)	3.95 ± 0.04	4.30 ± 0.06	0.000^***^
C/N	11.08 ± 0.11	10.06 ± 0.13	0.000^***^
δ^13^C (%0)	−11.66 ± 0.03	−11.61 ± 0.04	0.428
δ^15^N (%0)	−1.32 ± 0.23	−1.09 ± 0.30	0.541

Values are means ± standard error of the 26 hybrids. Levels of signification:

* P < 0.05;

*** *P < 0.001*.

The effect of phosphorous fertilization was also significant for the different leaf parameters studied. Thus, leaf total phosphorous content (P content) and chlorophyll content (LCC) strongly decreased in response to a lack of phosphorous fertilizer. The total nitrogen content (N) also decreased significantly (*P* < 0.000), although in a weaker manner, whereas the total carbon content (C) together with the C/N ratio increased slightly without phosphorous fertilizer, and the stable carbon and nitrogen isotopic composition did not change.

### The effect of phosphorous fertilization and the sensor altitude on vegetation indices

Phosphorous-input also affected the RGB and multispectral indices (Table 3). All RGB indices derived from aerial images were significantly affected by phosphorous fertilization except v^*^. For the RGB indices measured from the ground, only Hue, Saturation, a^*^, u^*^, GA and GGA were significantly affected. Regardless of how images were collected, GA and GGA exhibited the strongest changes, decreasing more than the half with the absence of phosphorous fertilization. In contrast, the CIE-XYZ color space indices, particularly a^*^ and u^*^, increased significantly in absence of phosphorous fertilization (*P* < 0.0001). Besides, the values of the vegetation indices varied significantly (*P* < 0.0001) with imaging height (ground vs. UAV), except for GA (ground/aerial; GA: NPF 0.08/0.07, OP 0.21/0.20; GGA: NPF 0.08/0.02, OP 0.20/0.12). Hue and GGA were lower when they were assessed on the ground rather than from the aerial platform, while the other indices showed the opposite behavior.

**Table 3:** Effect of phosphorous fertilization on remote sensing indices derived from RGB and spectral measurements within the non-phosphorous fertilized (NPF) and the optimal phosphorus (OP) conditions.

	**NPF**	**OP**	***p*-value**
**RGB INDICES/GROUND**			
*Intensity*	0.36 ± 0.00	0.36 ± 0.00	0.861
*Hue*	30.63 ± 0.45	39.34 ± 1.23	0.000^***^
*Saturation*	0.19 ± 0.00	0.18 ± 0.00	0.000^***^
*Lightness*	42.35 ± 0.11	42.67 ± 0.25	0.243
a^*^	1.18 ± 0.15	−1.93 ± 0.37	0.000^***^
b^*^	18.88 ± 0.23	18.48 ± 0.20	0.200
u^*^	10.82 ± 0.22	6.34 ± 0.49	0.000^***^
ν^*^	20.38 ± 0.24	20.65 ± 0.26	0.440
*GA*	0.08 ± 0.01	0.21 ± 0.01	0.000^***^
*GGA*	0.08 ± 0.00	0.20 ± 0.01	0.000^***^
**RGB INDICES/UAV**			
*Intensity*	0.50 ± 0.00	0.49 ± 0.00	0.003^**^
*Hue*	23.53 ± 0.37	29.64 ± 0.72	0.000^***^
*Saturation*	0.24 ± 0.00	0.22 ± 0.00	0.000^***^
*Lightness*	55.13 ± 0.25	53.94 ± 0.40	0.014^**^
a*	9.39 ± 0.22	4.42 ± 0.42	0.000^***^
b*	26.53 ± 0.22	25.18 ± 0.23	0.000^***^
u*	28.05 ± 0.34	19.54 ± 0.69	0.000^***^
ν*	28.28 ± 0.24	27.82 ± 0.25	0.192
*GA*	0.07 ± 0.01	0.20 ± 0.01	0.000^***^
*GGA*	0.02 ± 0.00	0.12 ± 0.01	0.000^***^
**SPECTRAL INDICES**			
*NDVI g*	0.30 ± 0.03	0.49 ± 0.03	0.000^***^
*NDVI*	0.35 ± 0.01	0.50 ± 0.01	0.000^***^
*SAVI*	0.16 ± 0.01	0.24 ± 0.01	0.000^***^
*OSAVI*	0.23 ± 0.01	0.34 ± 0.01	0.000^***^
*RDVI*	0.16 ± 0.00	0.25 ± 0.01	0.000^***^
*EVI*	0.22 ± 0.01	0.35 ± 0.01	0.000^***^
*PRI*	0.16 ± 0.01	0.18 ± 0.00	0.001^**^
*MCARI*	0.05 ± 0.04	0.06 ± 0.00	0.000^***^
*TCARI*	0.08 ± 0.00	0.09 ± 0.00	0.012^*^
*TCARI/OSAVI*	0.36 ± 0.01	0.26 ± 0.01	0.000^***^
*ARI2*	0.75 ± 0.02	0.67 ± 0.02	0.010^*^
*CRI2*	6.65 ± 0.12	6.03 ± 0.20	0.009^**^
*WBI*	0.92 ± 0.00	0.94 ± 0.01	0.000^***^

These indices are defined at section Material and Methods. Values are means ± SE of the individual values of the 26 genotypes. Levels of signification:

* P < 0.05;

** P < 0.01;

*** *P < 0.001*.

The multispectral index NDVI also decreased significantly (*P* < 0.0001) as response to lack of phosphorus fertilizer (Table 3). The values of NDVI were slightly lower when this index was measured with the hand-held sensor at ground level compared with the same index assessed from the multispectral camera placed in the aerial platform. Apart from EVI, which was not affected by phosphorus fertilization, the values of the other multispectral indices measured via the UAV's multispectral images (Table 1) were also significantly smaller (*P* < 0.000) in the absence of phosphorous fertilizer compared with optimum phosphorous.

Correlations between the remote sensing indices Hue, a^*^, u^*^, GA, GGA, and NDVI assessed at ground level against the same indices measured from the UAV were very strong (Table 4). Moreover, most of these indices exhibited a slope close to 1 (Supplementary Figure 2). In contrast, relationships reported for the remaining RGB indices (Intensity, Saturation, Lightness, b^*^, and v^*^) were much lower.

**Table 4:** Regression coefficients (r) of the relationships between the remote sensing indices measured at ground against the same VIs measured at aerial level.

	***r***	***p*-value**
*Intensity*	0.275	0.000^***^
*Hue*	0.902^***^	0.000^***^
*Saturation*	0.466	0.000^***^
*Lightness*	0.126	0.000^***^
*a*	0.919^***^	0.000^***^
*b*	0.316	0.000^***^
*u*	0.903^***^	0.000^***^
ν	0.310	0.000^***^
*GA*	0.970^***^	0.509
*GGA*	0.942^***^	0.000^***^
*NDVI*	0.889^***^	0.000^***^

Correlations were studied across plots within both trials conditions in combination. Levels of signification:

*** *P < 0.001*.

### Performance of remote sensing indices assessing grain yield and leaf phosphorous

Correlation coefficients for the relationships of grain yield with both the RGB (Table 5) and the multispectral indices (Table 6) were calculated. Within both phosphorus conditions and regardless of the imaging height (ground or from UAV) of data acquisition, GA and GGA were best correlated with grain yield, followed by Hue and a^*^. The u^*^ index also correlated well with grain yield but only when measured from the aerial platform. The rest of the RGB indices correlated far more weakly or did not correlate with grain yield, irrespective of the phosphorus fertilization status or the imaging height of index assessment. Combining both fertilization levels also gave similar results. The correlations of these indices against leaf phosphorus content within both phosphorus treatments were in general weak or absent. It was only under the combination of both fertilization levels that the remote sensing indices had a clearly improved correlation with leaf P concentration, particularly for the indices that exhibited the best correlations with grain yield. However, the correlations against P content were in all cases weaker than with grain yield.

**Table 5:** Regression coefficients of the relationships between the RGB-indices, measured at ground and aerial levels, with grain yield and P content.

	**Grain yield**			**P content**		
	**NPF**	**OP**	**Comb**.	**NPF**	**OP**	**Comb**.
**RGB INDICES/GROUND**						
*Intensity*	0.194	−0.217	−0.084	−0.014	−0.067	−0.041
*Hue*	0.777^***^	0.732^***^	0.827^***^	0.336	−0.370	0.594^*^
*Saturation*	0.468^*^	−0.027	−0.179	0.065	0.247	−0.429^*^
*Lightness*	0.459^*^	−0.014	0.205	0.086	−0.152	0.126
a^*^	−0.601^**^	−0.725^***^	−0.818^***^	−0.334	0.405^*^	−0.643^**^
b^*^	0.572^**^	0.226	0.171	0.110	−0.020	−0.157
u^*^	−0.300	−0.729^***^	−0.786^***^	−0.267	0.425^*^	−0.667^**^
ν^*^	0.642^***^	0.362	0.434^**^	0.151	−0.152	0.094
*GA*	0.816^***^	0.817^***^	0.878^***^	0.111	−0.369	0.707^**^
*GGA*	0.822^***^	0.816^***^	0.877^***^	0.122	−0.367	0.711^**^
**RGB INDICES/AERIAL**						
*Intensity*	−0.223	−0.715^***^	−0.620^***^	0.166	0.021	−0.359
*Hue*	0.731^***^	0.798^***^	0.868^***^	−0.062	−0.361	0.624^**^
*Saturation*	0.149	0.266	−0.235	−0.539^*^	−0.112	−0.581^*^
*Lightness*	−0.102	−0.653^***^	−0.526^***^	0.109	−0.047	−0.316
a^*^	−0.856^***^	−0.784^***^	−0.883^***^	−0.284	0.339	−0.750^**^
b^*^	0.192	0.002	−0.292^*^	−0.466^*^	−0.221	−0.575^*^
u^*^	−0.830^***^	−0.777^***^	−0.873^***^	−0.424^*^	0.284	−0.777^**^
ν^*^	0.318	0.084	0.016	−0.333	−0.337	−0.283
*GA*	0.837^***^	0.814^***^	0.891^***^	0.139	−0.343	0.693^**^
*GGA*	0.790^***^	0.752^***^	0.837^***^	0.206	−0.309	0.697^**^

Correlations were studied across plots within the non-phosphorus fertilization (NPF) and the optimal phosphorus (OP) trials, as well as both in combination (Comb.). Levels of signification:

* P < 0.05;

** P < 0.01;

*** *P < 0.001*.

**Table 6:** Regression coefficients of the relationships between the multispectral-indices and the multispectral with grain yield, P and N content.

	**Grain Yield**			**P Content**		
	**NPF**	**OP**	**Comb**.	**NPF**	**OP**	**Comb**.
**MULTISPECTRAL INDICES**						
*NDVI.ground*	0.734^***^	0.711^***^	0.863^***^	0.058	−0.423^*^	0.669^***^
*NDVI*	0.628^***^	0.643^***^	0.823^***^	0.324	−0.347	0.800^***^
*SAVI*	0.652^***^	0.644^***^	0.823^***^	0.159	−0.269	0.790^***^
OSAVI	0.657^**^	0.655^**^	0.829^***^	0.216	−0.303	0.797^***^
*RDVI*	0.658^***^	0.650^***^	0.829^***^	0.198	−0.286	0.795^***^
*EVI*	0.613^***^	0.529^**^	0.798^***^	0.119	−0.220	0.782^***^
*PRI*	0.039	0.312	0.406^**^	0.428^*^	0.032	0.466^*^
*MCARI*	0.358	−0.019	0.452^**^	−0.035	−0.033	0.463^*^
TCARI	0.172	−0.200	0.238	−0.147	0.055	0.314
TCARI/OSAVI	−0.401^*^	−0.618^**^	−0.748^***^	−0.368	0.283	−0.700^***^
*ARI2*	−0.012	0.286	−0.133	−0.286	−0.002	−0.363
*CRI2*	0.016	0.359	−0.091	−0.162	−0.064	−0.364
*WBI*	0.241	0.595^**^	0.598^***^	−0.014	−0.064	0.414^*^
**MULTISPECTRAL BANDS**						
B450	−0.348	−0.688^***^	−0.638^***^	−0.459	0.318	−0.383
B550	0.261	−0.505^**^	−0.102	−0.205	0.371	0.036
B570	0.032	−0.529^**^	−0.419^**^	−0.498^*^	0.212	−0.354
B670	−0.302	−0.566^**^	−0.739^***^	−0.540^*^	0.398	−0.731^***^
B700	−0.116	−0.525^**^	−0.602^***^	−0.463^*^	0.324	−0.567^**^
B720	0.269	−0.045	0.153	−0.319	0.125	0.047
B780	0.465^*^	0.477^*^	0.741^***^	−0.020	−0.122	0.688^***^
B840	0.496^*^	0.550^**^	0.779^***^	0.010	−0.137	0.744^***^
B860	0.442^*^	0.492^*^	0.753^***^	−0.051	−0.129	0.736^***^
B900	0.425^*^	0.537^**^	0.761^***^	−0.063	−0.083	0.739^***^
B950	0.390^*^	0.411^*^	0.724^***^	−0.024	−0.091	0.741^***^

Correlations were studied across plots within the non-phosphorus fertilization (NPF) and the optimal phosphorus (OP) trials, as well as both in combination (Comb.). Levels of signification:

* P < 0.05;

** P < 0.01;

*** *P < 0.001*.

Concerning NDVI, and regardless the fertilization level, the highest correlation with GY was found with ground spectroradiometer measurements, although the NDVI derived from the UAV was still highly correlated with GY (Table 6). Multispectral indices SAVI, RDVI, OSAVI, EVI, and WBI were also significantly correlated with GY within the two phosphorus conditions alone, or when both levels were combined. Individual multispectral bands presented significant correlations with yield, particularly under optimal phosphorus. Correlations of these indices with leaf P content were weak or absent, regardless of the phosphorus level, whereas spectral bands around 570, 670, and 700 nm significantly, but weakly, correlated with P content at the low fertilization level. In the case of the RGB indices, combining both treatments strongly increased the correlations between the multispectral indices and P content, particularly for the indices that best correlated with grain yield (NDVI, SAVI, RDVI, EVI, or OSAVI). However, the strengths of the correlations were always lower than for grain yield.

For the purpose of testing how the combination of different indices measured from the aerial platform may improve the strength and accuracy of the assessment of grain yield and phosphorous concentration, stepwise regressions were performed (Table 7). The best predictive equations of grain yield were achieved using RGB indices, which were the most significant measurements in the absence of phosphorous fertilizer. The multispectral bands and indices performed better at predicting grain yield under optimum phosphorus conditions than the non-fertilized conditions. In contrast, the prediction of P was not as good as GY and the only significant equations were found at the non-phosphorous fertilization conditions (*P* < 0.050).

**Table 7:** Multilinear regression (stepwise) of grain yield (GY) as dependent variable and the different categories of remote sensing traits (RGB VIs, multispectral VIs, and specific multispectral bands) measured from the unmanned aerial vehicle within the non-phosphorus fertilization (NPF) and the optimal phosphorus (OP) trials.

			**Equation**	***R*^2^**	**RSE**	***p*-value**	**Portion of variance**
GY	*NPF*	Aerial RGB VIs	GY = −0.25·u*+ 13.99·GA + 11.65	0.821	0.590	0.000	u* = 0.49
							*GA = 0.50*
		Multispectral VIs	*GY = 59.08·MCARI – 12.46·TCARI/OSAVI + 7.38*	0.463	0.769	0.000	*MCARI* = 0.46
							*TCARI/OSAVI* = 0.53
	*OP*	Aerial RGB VIs	*GY = 12.31·GA + 5.00*	0.662	0.596	0.000	*GA* = 1.00
		Multispectral VIs	*GY = −43.94·NDVI + 189.93·RDVI – 59.62·EVI + 3.36*	0.652	0.632	0.000	*NDVI* = 0.31
							*RDVI* = 0.40
							*EVI* = 0.28
P content	*NPF*	Aerial RGB VIs	P content = −0.26·Hue – 0.49·a*+ 13.00	0.436	0.337	0.001	*Hue = 0.41*
							a* = 0.58
		Multispectral VIs	*P content = −146.66·NDVI – 995.36·SAVI + 1289·RDVI + 0.53*	0.311	0.381	0.038	*NDVI* = 0.39
							*SAVI* = 0.29
							*RDVI* = 0.31
	*OP*	Aerial RGB VIs	P content = 0.47· b* – 0.56·ν*+ 8.82	0.210	0.520	0.065	b* = 0.34
							v* = 0.65
		Multispectral VIs	*P content = 77.16· SAVI – 86.16·RDVI + 7.20*	0.151	0.539	0.150	*SAVI* = 0.46
							*RDVI* = 0.53

*R^2^, determination coefficient; RSE, Residual Standart Error*.

In order to check the ability of the remote sensing indices to predict genotypic differences in yield, we correlated the genotypic values of the different categories of remote sensing traits evaluated in the seedlings with the yield of each hybrid determined from multi-location trials developed in parallel (Table 8). Every index that correlated with yield in our experiment, in either the absence of phosphorous fertilizer or in optimum conditions, also showed significant correlations with the genotypic yield data of the multilocation study. The correlation coefficients calculated with the RBG and the multispectral indices against the yield of the multilocation study were very similar to those found between these indices and the grain yield in the present study. The best correlated RGB VI's were GA and GGA again, both ground and aerial measurements. Also, the spectral indices associated with the greenness and density measurements correlated greatly with the genotypic yield, and to a similar extent as the correlation with grain yield in the same trials. On the other hand, the RDVI and the WBI correlated even better with grain yield from the multilocation trials than with the grain yield of the present remote sensing trial.

**Table 8:** Regression coefficients (r) of the relationships across the genotypes of the VI's measured in seedlings at non-phosphorus fertilization (NPF) and optimal phosphorous (OP) conditions in this study against grain yield data from other trials.

	**NPF**	**OP**
**RGB INDICES/GROUND**		
*Intensity*	0.079	−0.237
*Hue*	0.494^*^	0.695^***^
*Saturation*	0.562^**^	−0.039
*Lightness*	0.311	−0.047
a^*^	−0.232	−0.677^***^
b^*^	0.592^**^	0.187
u^*^	0.057	−0.685^***^
ν^*^	0.602^**^	0.314
*GA*	0.738^***^	0.830^***^
*GGA*	0.741^***^	0.828^***^
**RGB INDICES/UAV**		
*Intensity*	−0.465^*^	−0.643^***^
*Hue*	0.767^***^	0.766^***^
*Saturation*	0.491^**^	0.360
*Lightness*	−0.317	−0.570^**^
a^*^	−0.705^***^	−0.721^***^
b^*^	0.423^*^	0.137
u^*^	−0.625^***^	−0.692^***^
ν^*^	0.450^*^	0.209
*GA*	0.848^***^	0.779^***^
*GGA*	0.785^***^	0.730^***^
**SPECTRAL INDICES**		
NDVI g	0.752^***^	0.594^**^
NDVI	0.656^***^	0.629^***^
*PRI*	−0.207	0.223
*SAVI*	0.658^***^	0.630^***^
MCARI	0.399^*^	−0.017
*WBI*	0.486^*^	0.573^**^
RDVI	0.721^***^	0.630^***^
*EVI*	0.403^*^	0.334
*ARI2*	0.133	0.162
*CRI2*	0.112	0.243
*TCARI*	0.304	−0.157
*OSAVI*	0.552^**^	0.611^***^

Levels of signification:

* P < 0.05;

** P < 0.01;

*** *P < 0.001*.

## Discussion

### Phosphorus fertilization effect on grain yield

Phosphorous is an essential nutrient for plant growth and development (Manschadi et al., 2014). For that reason, the yield of the hybrids was strongly affected by the lack of phosphorus fertilizer, and leaf phosphorous content correlated with grain yield across hybrids in the non-phosphorus-fertilized trial. The large variability in plant performance across the hybrids that was revealed in our results presumably reflects differences in P use efficiency as well as genotypic differences in yield potential (i.e., productivity under optimal agronomical conditions). In general, most reports state that P deficiency reduces photosynthetic capacity and efficiency through different mechanisms (Brooks et al., 1988).

Yield variations caused by differences in the water status of the plants can be ruled out through the lack of differences in δ^13^C. Even for a C4 plant like maize, differences in plant water status, and intrinsic photosynthetic metabolism may be reflected in the δ^13^C of the plant matter, with δ^13^C decreasing in response to water stress (Farquhar et al., 1989; Monneveux et al., 2007). We did not found differences in δ^13^C associated to fertilization. In contrast, significant differences between the two fertilization conditions were detected in the WBI values. This index uses the reflectance spectra at the near and far-infrared region as an indication of water absorption. Hence, higher WBI values indicate a better water status. Optimal growing conditions had enabled faster seedling growth and therefore turgid leaves (i.e., more watered), although past studies have also indicated that WBI can predict the leaf area index (Roberts et al., 1998; Qiu et al., 2007). Thus, higher WBI values at optimum P conditions must be due to a larger canopy area rather than water status differences. Nevertheless, some reports have indicated that phosphorus fertilization can help crops to use water more efficiently under limited moisture conditions (Waraich et al., 2011).

Phosphorous and nitrogen content in the leaves correlated within each fertilization levels (Supplementary Figure 3) and both contents were higher under optimal compared with non-phosphorous fertilization conditions. Differences in nitrogen content may account for the variation across genotypes and fertilization levels in LCC and the fact that at least under NPF chlorophyll content and phosphorous content correlated positively.

### Comparative performance of ground vs. aerially assessed indices at determining genotypic differences in grain yield

**Figure 2:**
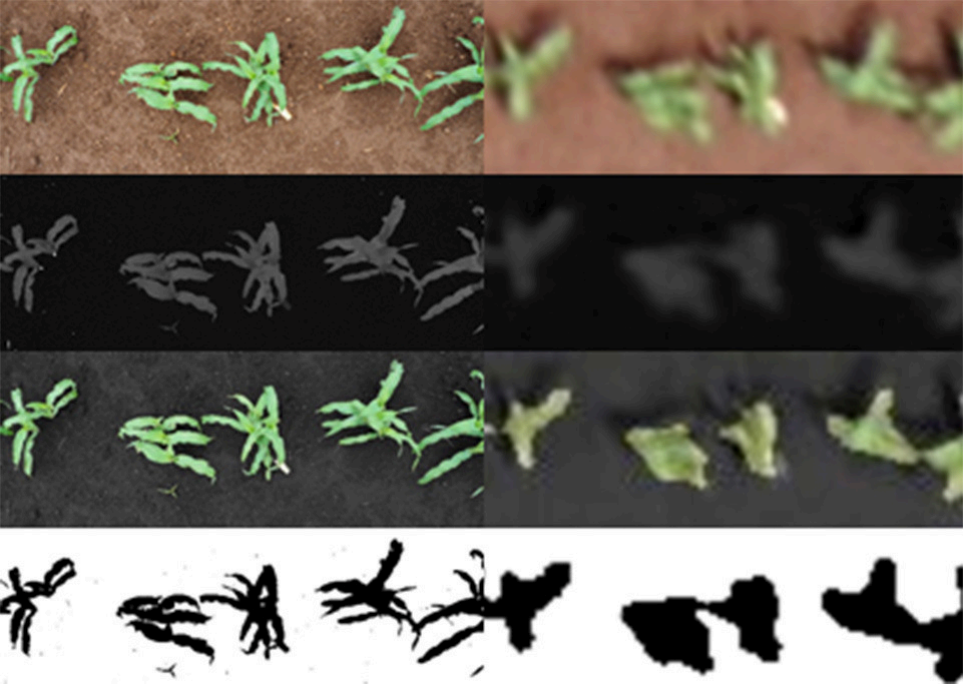
Examples of the differences in resolution between images taken at ground level and aerially.

The vegetation indices derived from conventional digital RGB images have been proposed as a means of estimating green biomass and grain yield in maize and other cereals under stress conditions (Ahmad and Reid, 1996). As the ground and aerial measurements were taken at the same time on the same day, variation in environmental variables such as light intensity and brightness can be almost negligible. Thus, the main differences are due to the resolution of the pictures (Figure 2). Besides using cameras with the same sensor size (17.3 × 13 mm) that capture images at the same resolution (16-megapixels), the final resolution of the images was also affected (by the square of) the distance between the camera and the object (in this case the plots). While the images collected in our study from the UAV only reached a resolution of 488 × 193 pixels per plot, the spatial resolution of the images taken from the ground was 4,608 × 3,072 pixels per plot. When the spatial resolution is very high, plants in the image are well-defined; however, when the spatial resolution is poorer, the boundaries between plants and soil are fuzzy, and consequently, there is usually a higher portion of pixels including information of both vegetation and bare soil (Torres-Sánchez et al., 2014). Despite such differences in resolution, some indices like a^*^ and u^*^ assessed aerially correlated better against grain yield, whereas others exhibited similar performance to ground determined indices, except for GGA which correlated slightly weaker when assessed from the aerial platform. The a^*^ and u^*^ indices are more likely to reflect color components that are more sensitive to the scene's illumination and the camera's self-adjustments (Casadesús et al., 2005), thus being more limited by soil lightness and therefore performing better at the aerial level with reduced image resolution. Conversely, a reduction in the number of pixels in the image makes it more difficult to identify differences in vegetation color, so GGA performed better at ground level. Nevertheless, advances in digital photography allow sufficiently high resolution for low-altitude aerial imaging to be a viable and economical monitoring tool for agriculture (Sankaran et al., 2015). Moreover, aerial photographs enable coverage of the whole plot (which usually is not the case for images taken at ground level) and therefore, to some extent, may compensate for the loss of spatial resolution. In this sense, correlations with grain yield by indices derived from aerial imaging were generally only slightly weaker than indices measured at ground level. Some of the RGB indices like Hue, a^*^, u^*^, GA, or GGA and the NDVI, produced correlation coefficients higher than *R*^2^ = 0.900 when compared to the same indices measured at ground level and from the aerial platform (Supplementary Figure 2). This is despite the methodological differences between index determination at ground level (on an individual plot basis) and the aerial platform (across a whole trial and further segmented into individual plots). Therefore, both approaches are able to offer essentially similar kinds of information.

### Comparative performance of the RGB vs. multispectral indices at determining genotypic differences in grain yield

The RGB-based indices, GA and GGA, were the best at GY prediction, outperforming other RGB indices, NDVI and the rest of the spectral indices. Considering that the data of our study was collected at an early phenological stage, the plants were not able to cover the soil completely. Therefore, the superior performance of these indices should be attributable, at least in part, to their insensitivity to soil color (Casadesús et al., 2007). GA quantifies the portion of green pixels to the total pixels of the image and is a reliable estimator of vegetation cover (Lukina et al., 1999). By contrast, GGA does not incorporate the yellowish green fraction of vegetation when the GA becomes saturated during late phenological periods. Therefore, elevated GA and GGA indices, probably driven by a higher biomass, seem to be more relevant for predicting higher yield. Although these indices performed in a very similar way at both measurement locations, when the GGA was measured at ground level it tended to be more highly correlated to GY. Besides other considerations the far higher resolution of the RGB compared with the multispectral images may be also relevant when working from an aerial platform.

A recent study has concluded that RGB images performed better than NDVI in determining genotypic differences in hybrid maize yield under different nitrogen fertilization conditions (Vergara-Díaz et al., 2016). The results of our research include the NDVI and its reformulations as the SAVI, OSAVI, EVI, and RDVI indices, which were best correlated with GY. These indices, which are based on the strong contrast between the near infrared (NIR) and (R) bands, are optical measurements of canopy greenness and canopy cover (Tucker, 1979). NDVI is a widely accepted approximation for assessing crops under different growing conditions, but it can fail to distinguish changes in soil cover and plant density from changes in vegetation color (Steven et al., 1996). As our study was made at an early stage of development, the plants did not have enough biomass to cause this saturation problem. The SAVI was developed as a modification of the NDVI, to correct the brightness incidence of the soil (Huete, 1988). Notwithstanding the reduction in soil noise problems, correlations of the SAVI with GY were not improved in comparison to the NDVI. The optimization of this index, which applied an adjusting coefficient (Rondeaux et al., 1996) that resulted in the OSAVI, also did not improve the correlation with GY, but rather caused the opposite. The RDVI and the EVI are another indices based on the NDVI, which have been developed with the intention of correcting the rapid saturation due to dense vegetation (Liu and Huete, 1995). Even though this was not a problem in our study, the fact that those indices emphasize the vigor of vegetation has enabled achieving quite strong correlations, similar to NDVI.

MCARI is an index that measures the depth of chlorophyll absorption at 670 nm relative to the reflectance at 550 and 700 nm (Daughtry, 2000). TCARI is a transformation developed to counteract the effect of soil background (Haboudane et al., 2002). However, both indices are still sensitive to the background reflectance properties. The plots studied were particularly characterized by a low leaf area index, so neither the MCARI nor the TCARI were adequate for our experiment. Anthocyanin and carotenoid pigments were also detected by the ARI2 and the CRI2 indices, but no valuable information has been obtained.

The complementary metal-oxide-semiconductor (CMOS) image sensor of the micro-MCA12 camera is optimized to collect wavelengths at ~800 nm, dropping in a smooth curve to a low relative efficiency at 400 nm in the visible wavelengths and a smaller reduction in efficiency at 1050 nm in the NIR, at the limits of its range. As a consequence, the efficiency of the measurements in the blue band (450 nm) is considerably lower (20%) in comparison to the measurements of the NIR or the R bands (85% both). Due to this limitation in the blue region sensitivity, more noise is included in the measurements of the blue band. Moreover, inadequate phosphorus content can result in a darkening of the leaves to a purple color. This would explain why the single band measurement in the blue region correlated with GY at optimum conditions but it failed to do so under non-fertilized conditions. The correlation analysis between each multispectral band and yield has identified sensitive wavelengths under both phosphorus levels, and this ranges from 780 to 950 nm of the near-infrared (NIR, 750–1,350 nm) region of the spectrum.

The results obtained proved that measurements at an early growing date, while the plants are still seedlings, are optimal for the assessment of the future yield.

### Performance of RGB and multispectral indices at determining genotypic differences derived from leaf phosphorus concentration

The strength of the correlations inside each treatment between the indices and the P content were far lower than of these indices with GY. Distribution of values is not uniform and in fact the linear correlation has not any sense besides to show these vegetation indices are able to clearly differentiate between the two different groups of phosphorous fertilization (but not across genotypes within each fertilization level). The same happened with the LCC and the leaf nitrogen content (Supplementary Figure 3). The two different fertilization levels caused differences in leaf phosphorous content but indirectly also differences in leaf chlorophyll and total nitrogen contents (and at that with an abundance of N fertilizer applied to both treatments). Therefore, differences in leaf color between treatments are evident (less chlorophyll and nitrogen content in the leaves on non-phosphorous fertilized plants). However, similar to a^*^, GA, and GGA (Supplementary Figure 4), leaf chlorophyll and nitrogen contents did not correlate or just did marginally (SPAD values within NPF) against leaf phosphorous content. Again, the differences between fertilization levels accounted for the significant relationship of leaf chlorophyll and N contents against leaf phosphorous content when data of both fertilization levels were combined. Moreover, there is a lack of consistency between the ground and aerial RGB index correlations in regard to phosphorous content (Table 5). In contrast, the correlations with grain yield follow the same patterns for both fertilization levels. Therefore, the significance of the correlations of the indices with phosphorus concentration may be related to the relationship between leaf phosphorus concentration and green biomass due to phosphorous is an essential element in plant growth (Manschadi et al., 2014; Gemenet et al., 2016). Indices better assessed differences in leaf phosphorous concentration at the low phosphorous conditions compared to optimum conditions due to the primary capacity of these indices to strongly correlate with green biomass and thus grain yield.

Similarly, the multispectral indices didn't show significant correlations with P content within each fertilization level, while several of these indices correlated with GY. Only the PRI correlated with leaf phosphorous content (and just under low P conditions). The PRI is a spectral index increasingly used as an indicator of photosynthetic efficiency because it is based on the short-term reversible xanthophyll pigment cycle (Peñuelas et al., 2011). Low phosphorus levels can lead to an increase in the de-epoxidation process, which augments the relative amount of zeaxanthin and decreases violaxanthin (Goodwin, 1980; Tambussi et al., 2002). Zeaxanthin is essential for dissipation of excess energy as heat in chloroplasts (Demmig-Adams et al., 2013). The weak but still significant correlations between the PRI and the P content suggest a similar photoprotection response. In other studies, similar findings have been reported that associate nutrient deprivation with increased zeaxanthin levels and thus lower PRI values (Filella et al., 1996). In reference to the multispectral bands, only the bands located at 570, 670, and 700 nm correlated with the leaf phosphorous content, and these were a weakly correlation with the leaf phosphorous content. These bands correspond to the green (570 nm) and red regions (670 and 700 nm) and they have been used to assess non-stressed vegetation (Thenkabail et al., 2002). Higher values of reflection at these bands might correspond to vigorous plants with higher P content. These results are in conflict with the results obtained by Osborne et al. (2002), who reported a significant spectral response in the NIR region to the P concentration in corn.

## Conclusions

There is a need for phenotyping tools which increase the selection efficiency and to understand mechanisms of phosphorous tolerance. This study clearly shows a genotypic variability for low phosphorous tolerance, with a reduction in yields of 25% in average in comparison with the optimum conditions. Previous studies in the literature suggests that only when reduction is 75% or more, selecting for specific adaptation to tolerance to low nutrient availability is the strategy (Bänziger et al., 1997; Masuka et al., 2012). However, selecting for yield potential instead than for specific adaptation to low phosphorous, still makes sense when the yield reduction associated was moderate, like in this study, which is the usual situation in agronomical scenarios. Hence, indices also correlated with the yield of the hybrids when they were performed under the high yielding conditions.

This study emphasizes the capabilities of RGB vegetation indices as phenotypic traits for predicting maize performance during early stages of crop growth. GA was the vegetation index best correlated with grain yield across maize hybrids and regardless the phosphorous fertilization level and therefore this index may serve to select the most productive hybrids for the SSA. RGB indices assessed at ground level were comparable to those measured from an aerial platform. Moreover, RGB indices performed better than multispectral vegetation indices. The use of vegetation indices derived from RGB images may represent a very affordable approach for phenotyping and may become even more economical due to the similarity between results obtained from ground evaluation and those achieved from aerial platforms. The phenotypic correlations found between the remote sensing indices of seedlings and the genotypic yield data collected in the multi-location trials confirm their usefulness. Despite its comparatively low tech and low-cost nature, digital photography is a promising approach, and its derived indices have demonstrated potential for the assessment of crop management in maize, making it ideal for developing countries in particular.

Additionally, RGB-derived vegetation indices are also amenable for monitoring the effects of phosphorous fertilizer applications. However, only some of the indices best correlated with grain yield exhibited significant, albeit weaker, correlations with leaf phosphorus content. Moreover, these correlations were only present under low phosphorus fertilization, which suggests that they were linked to differences in biomass and grain yield caused by phosphorous fertilization.

## Author contributions

MZ-A, BP, and JC managed and directed the maize trials at the Southern Africa regional office of CIMMYT in Harare, Zimbabwe. SK carried out the UAV flights for the obtainment of aerial measurements. OV-D and JA conducted the field measurements and the collection of samples. AG-R processed the images, analyzed the samples and wrote the paper under the supervision of JA and SK and with the contributions from all the other authors.

### Conflict of interest statement

The authors declare that the research was conducted in the absence of any commercial or financial relationships that could be construed as a potential conflict of interest.

## Abbreviations

SSA: Sub-Saharan Africa
RGB: Red-Blue-Green
NDVI: Normalized Difference Vegetation Index
UAV: Unmanned aerial Vehicle
GY: Grain yield
VIs: Vegetation Indices
HIS: Hue-Intensity-Saturation
GA: Green Area
GGA: Greener Area
AN: Ammonium Nitrate
CIMMYT: International Maize and Wheat Improvement Center
masl: meters above sea level
CP-OES: Inductively Coupled Plasma Optical Emission Spectroscopy
P content: Phosphorous content
LCC: Chlorophyll Content
PRI: Photochemical Reflectance Index
SAVI: Soil Adjusted Vegetation Index
MCARI: Modified Chlorophyll Absorption Ratio Index
WBI: Water Band Index
RDVI: Renormalized Difference Vegetation Index
EVI: Enhanced Vegetation Index
ARI2: Anthocyanin Reflectance Index 2
CRI2: Carotenoid Reflectance Index 2
TCARI: Transformed Chlorophyll Absorption in Reflectance Index
OSAVI: Optimized Soil-Adjusted Vegetation Index
ΔF/Fm′: Effective Fluorescence Quantum yield
NIR: Near-Infrared.
