# Supplementary material for: Comparative Performance of Ground vs. Aerially Assessed RGB and Multispectral Indices for Early-Growth Evaluation of Maize Performance under Phosphorus Fertilization
Source: Front Plant Sci. 2017 Nov 27;8:2004. doi: 10.3389/fpls.2017.02004 (PMC5711853; doi:10.3389/fpls.2017.02004)
Supplement: Supplementary file 1 [file DataSheet1.DOCX]

Supplementary Material

Comparative performance of ground versus aerially assessed RGB and multispectral indices for early-growth evaluation of maize performance under phosphorus fertilization

Adrian Gracia-Romero, Shawn C. Kefauver, Omar Vergara-Díaz, Mainassara A. Zaman-Allah, Boddupalli M. Prasanna, Jill E. Cairns and José L. Araus*

*** Correspondence:** José L. Araus: jaraus@ub.edu

# Supplementary Figures

**
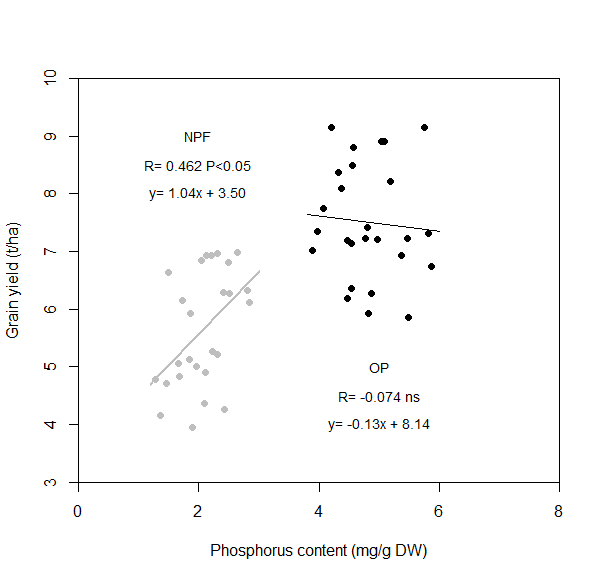
**

**Supplementary Figure 1.** Relationship of grain yield to leaf phosphorous content within both fertilization conditions. Gray dots correspond to the non-phosphorous fertilization conditions, while the black dots correspond to the optimum phosphorous conditions.


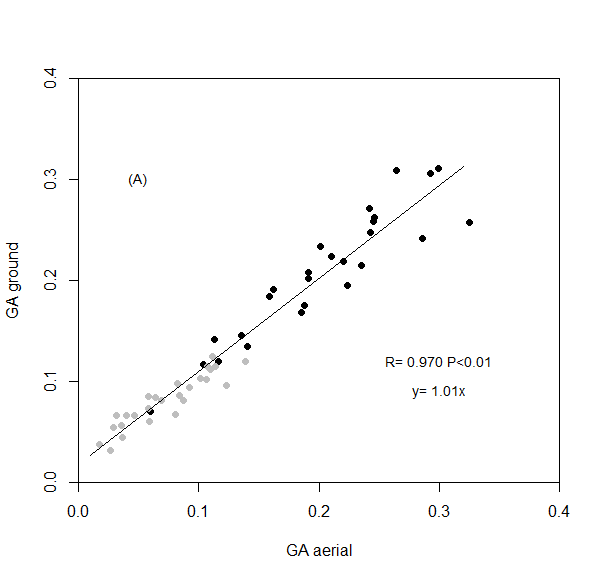


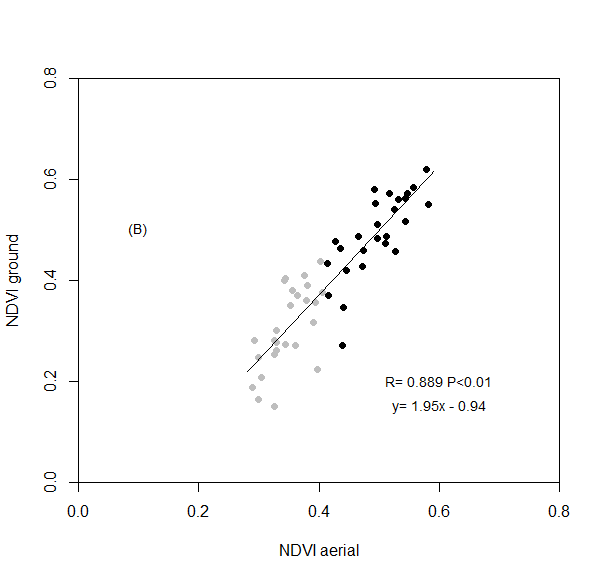


**Supplementary Figure 2.** Correlations between measurements at ground level and those collected aerially levels for the RGB index GA (A) and the multispectral index NDVI (B). Gray dots correspond to the non-phosphorous fertilization conditions, while the black dots correspond to the optimum phosphorous conditions.


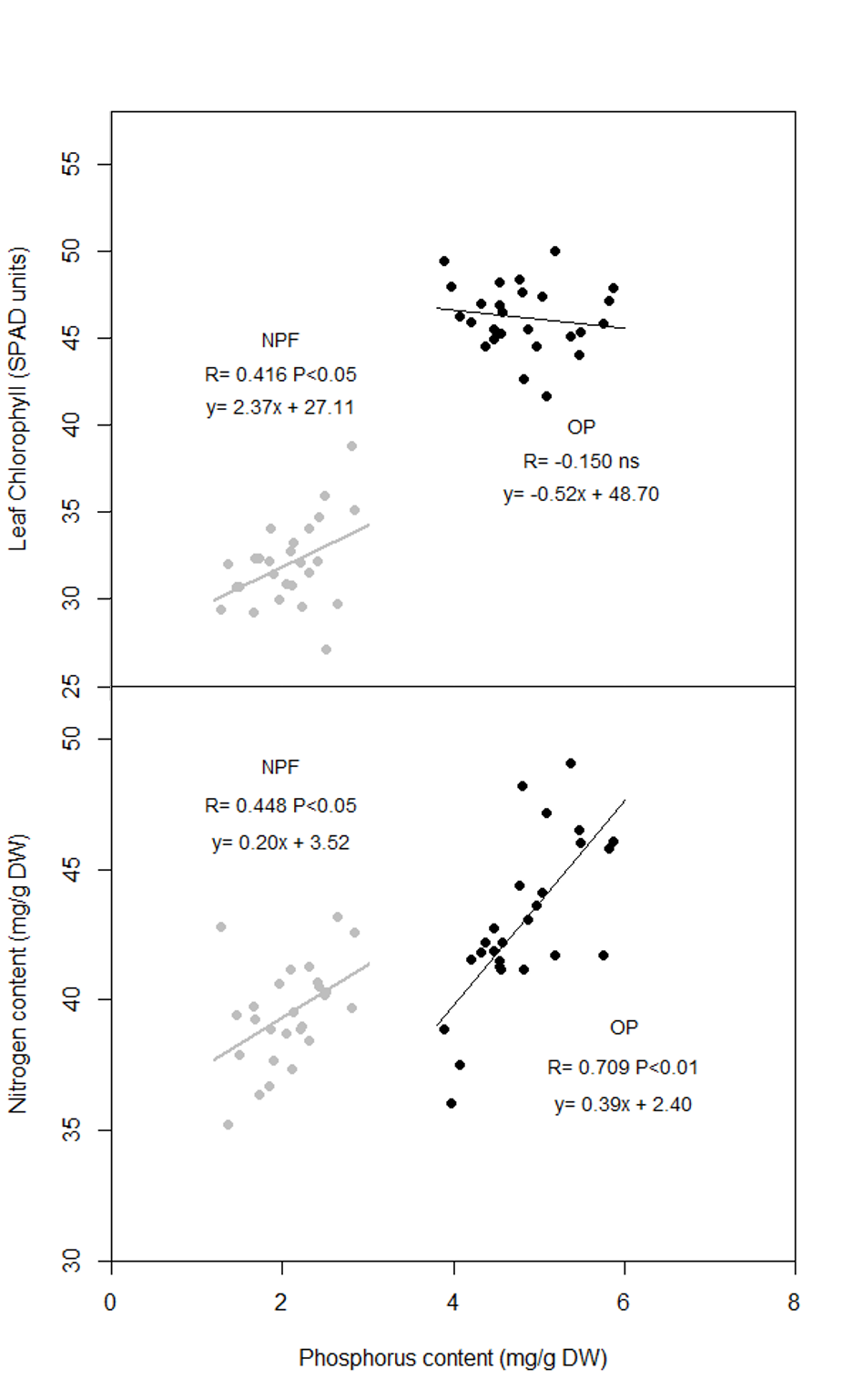


**Supplementary Figure 3.** Relationship of chlorophyll measurements and the nitrogen content to leaf phosphorous content within both fertilization conditions. Gray dots correspond to the non-phosphorous fertilization conditions, while the black dots correspond to the optimum phosphorous conditions.


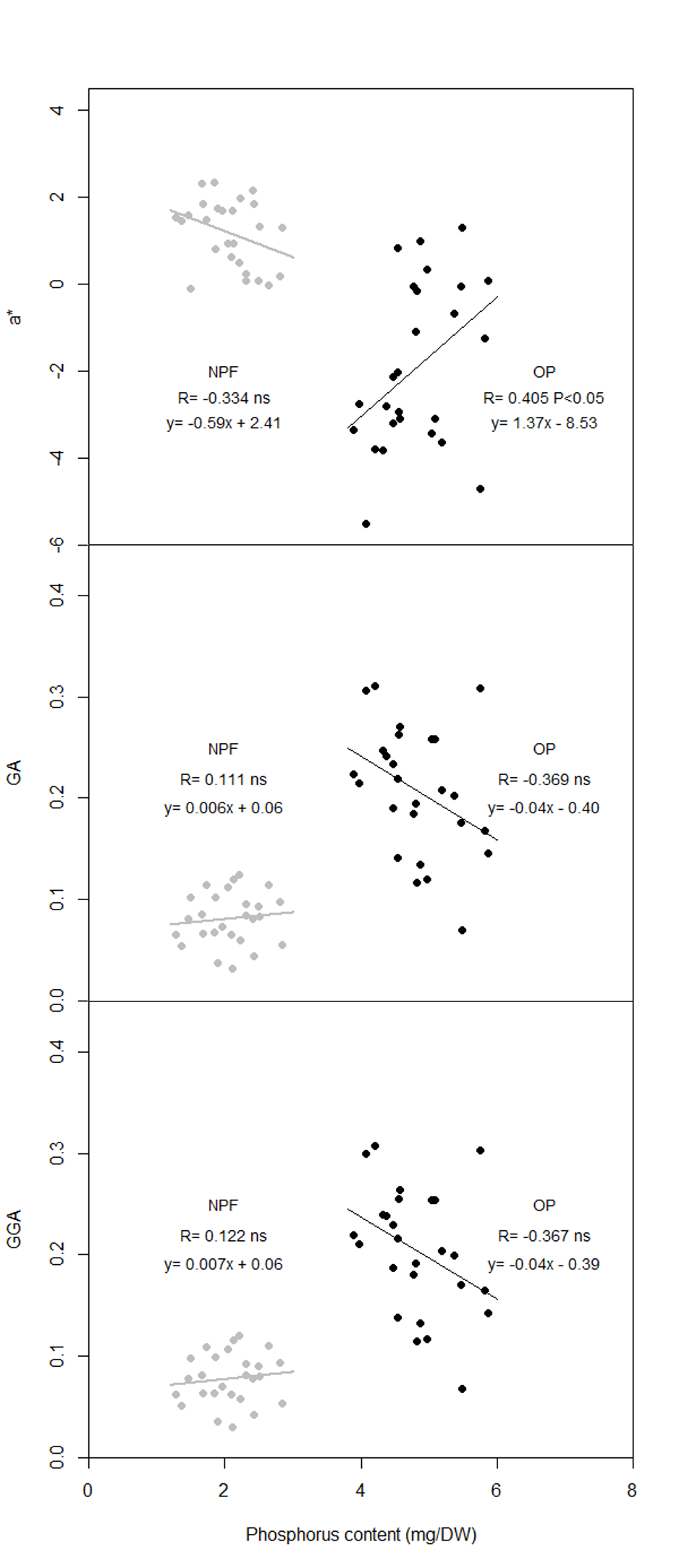


**Supplementary Figure 4.** Relationship of the RGB indices a*, GA and GGA to leaf phosphorous content within both fertilization conditions. Gray dots correspond to the non-phosphorous fertilization conditions, while the black dots correspond to the optimum phosphorous conditions.
